# A pangenomic analysis of the *Nannochloropsis* organellar genomes reveals novel genetic variations in key metabolic genes

**DOI:** 10.1186/1471-2164-15-212

**Published:** 2014-03-19

**Authors:** Shawn R Starkenburg, Kyungyoon J Kwon, Ramesh K Jha, Cedar McKay, Michael Jacobs, Olga Chertkov, Scott Twary, Gabrielle Rocap, Rose Ann Cattolico

**Affiliations:** 1 Bioscience Division, Los Alamos National Laboratory, Los Alamos 87545, NM, USA; 2 Department of Molecular and Cell Biology, University of California-Berkeley, Berkeley 94720, CA, USA; 3 School of Oceanography, University of Washington, Seattle 98195, WA, USA; 4 Biology Department, University of Washington, Seattle 98195, WA, USA

**Keywords:** *Nannochloropsis*, Chloroplast, Mitochondria, Genome, Stramenopiles, Genome evolution, Gene divergence

## Abstract

**Background:**

Microalgae in the genus *Nannochloropsis* are photosynthetic marine Eustigmatophytes of significant interest to the bioenergy and aquaculture sectors due to their ability to efficiently accumulate biomass and lipids for utilization in renewable transportation fuels, aquaculture feed, and other useful bioproducts. To better understand the genetic complement that drives the metabolic processes of these organisms, we present the assembly and comparative pangenomic analysis of the chloroplast and mitochondrial genomes from *Nannochloropsis salina CCMP1776.*

**Results:**

The chloroplast and mitochondrial genomes of *N. salina* are 98.4% and 97% identical to their counterparts in *Nannochloropsis gaditana.* Comparison of the *Nannochloropsis* pangenome to other algae within and outside of the same phyla revealed regions of significant genetic divergence in key genes that encode proteins needed for regulation of branched chain amino synthesis (acetohydroxyacid synthase), carbon fixation (RuBisCO activase), energy conservation (ATP synthase), protein synthesis and homeostasis (Clp protease, ribosome).

**Conclusions:**

Many organellar gene modifications in *Nannochloropsis* are unique and deviate from conserved orthologs found across the tree of life. Implementation of secondary and tertiary structure prediction was crucial to functionally characterize many proteins and therefore should be implemented in automated annotation pipelines. The exceptional similarity of the *N. salina* and *N. gaditana* organellar genomes suggests that *N. gaditana* be reclassified as a strain of *N. salina*.

## Background

Stramenopiles encompass a broad array of golden brown algae that are morphologically diverse, ranging from unicells (e.g., diatoms) to large bladed species (e.g., kelps). These organisms acquired their chloroplast via secondary endosymbiosis, thus their evolutionary progression differs significantly from that of their green (*Chorophyta*) and red (*Rhodophyta*) primary endosymbiotic algal counterparts [1]. Among the 17 classes of stramenopiles, the *Eustigmatophyceae* represent one of the smallest divisions. Members of this class, found in fresh, brackish, and marine waters, are minute in size, coccoid in shape, yellow-green in color, and essentially indistinguishable from one another given the lack of defining morphological characteristics [2].

Select unicellular photosynthetic microalgae have been targeted for commercial applications given their ability to efficiently accumulate biomass and/or lipids for conversion into renewable transportation fuels and other useful bioproducts. Algae within the Eustigmatophyceae, specifically within the genus *Nannochloropsis* are actively being evaluated for use in biofuel and aquaculture production systems due to their ability to convert a significant portion of their biomass (up to 60% dry weight) into lipids [3-5]. Although significant effort has been expended to characterize growth phenotypes and the fatty acid content within the genus *Nannochloropsis*[6-11], knowledge of the genetic and genomic basis that defines and controls their physiological behavior are still lacking; critical information required to support effective genetic engineering strategies. Recently, an analysis of the mitochondrial and chloroplast genomes of seven strains from six species of *Nannochloropsis* revealed that the genomic content was highly conserved between these species yet, evolutionarily divergent ‘hotspots’ were present, enabling an accurate phylotyping of these closely related species [12].

Here, we present the first analysis of the chloroplast and mitochondrial genomes from *N. salina CCMP1776* and the resequencing and analysis of *N. oculata* CCMP525. To determine the unique features of these *Nannochloropsis* organelles, we compared these genomes to the complete organellar genomes of *Nannochloropsis gaditana* CCMP526 [13], an improved draft assembly and annotation of *Nannochloropsis oceanica str.* LAMB0001 [14], and to the six strains analyzed by Wei, et al. [12]. Through these analyses, genomic variations and similarities were identified between *Nannochloropsis* and its stramenopile relatives. Striking similarity was observed between the organellar genomes of *N. salina* and *N. gaditina*. Additionally, novel modifications to key metabolic genes in the organelles of the genus *Nannochloropsis* were uncovered which further inform the physiological properties of this unique algal taxon.

## Methods

### Culturing and DNA purification

*Nannochloropsis salina* (CCMP1776) was grown at 30°C with a modified F/2-Si media with 10X nitrate and 7X phosphate [15] utilizing fluorescent plant grow lights at 1200 μEm^-2^ s^-1^ on a 16/ 8 hour light dark cycle. Dissolved O_2_ was maintained at 100% of base level from an 80% N_2_/ 20% O_2_ atmosphere through mass flow regulation of N_2_ or O_2_ gas input. Cell cultures were maintained at pH 8.2 utilizing pH controlled mass flow valves supplementing CO_2_ as needed into the continuous air supply. Optical density was continually monitored utilizing a Bugeye reading at 850 nm and cells were harvested during late log growth by centrifugation. Genomic DNA was isolated and purified utilizing the Qiagen DNeasy plant maxi kit. Cells were lysed by extraction in the Avestin Emulsiflex-B15 homogenizer at 30,000 psi prior to purification.

*Nannochloropsis oculata* (CCMP 525) was axenically maintained in 2.8 L wide-mouth Fernbach flasks that contained 1,000 ml F/2 medium [15]. The flasks were plugged with cheesecloth-covered, hand rolled cotton stoppers and capped with #2 Kraft autoclave bags (Paper Mart, Orange, CA.). Cultures were maintained at 20°C on a 12 h light: 12 h dark photoperiod at 100 μEm^-2^ s^-1^ light intensity using full spectrum T12 fluorescent light bulbs (Pacific Lamp Supply Co., Seattle, WA.). Cell counts were accomplished using an Accuri C6 flow cytometer (BD Scientific, Ann Arbor, MI). Cultures were harvested at early stationary phase of growth and total high molecular weight DNA (greater than 500 kb in size) was extracted from *N. oculata* using the Qiagen Genomic-Tip 500G kit according to manufacturer’s directions (Qiagen, Valencia, CA, USA).

### Sequencing and assembly

*N. salina* chloroplast and mitochondrial genomes were sequenced using a combination of Illumina [16] and 454 sequencing technologies [17]. A 1 X 100 base pair shotgun library was prepared using standard TruSeq protocols and sequenced from bulk *N. salina* genomic DNA on an Illumina HiSeq2000 sequencer, generating approximately 100 million reads. Additional shotgun single-end and paired-end (11 kb insert) DNA libraries were prepared for sequencing on the 454 Titanium platform, generating 0.807 million and 3.23 million reads, respectively. The 454 single-end data and the 454 paired end data (insert size 4720 +/- 1180 bp) were assembled together using Newbler, version 2.3 (release 091027_1459). The Illumina-generated sequences were assembled separately with VELVET, version 1.0.13 [18]. The resulting consensus sequences from both the VELVET and Newbler assemblies were computationally shredded into 10 kb fragments and were re-assembled with reads from the 454 paired end library using parallel phrap, version 1.080812 (High Performance Software, LLC). The chloroplast and mitochondrial replicons were identified in this final hybrid assembly based on: a) increased coverage in the 454 paired-end library (> 20 times higher than nuclear genome reads), b) the absence of paired end links to other contigs in assembly and, c) verification via homologous blast searches against the *N. gaditana* chloroplast and mitochondrial genomes. Sequence reads that belonged to each respective organelle were removed from the main project and re-assembled separately. Mis-assemblies in the contigs/scaffolds were corrected using gapResolution (Cliff Han, unpublished script) or Dupfinisher (Han, 2006) and repeat resolution was performed in Consed to generate the final circular consensus sequence. The final, fully assembled chloroplast genome was supported by > 500x average coverage from both sequencing platforms.

*N. oculata* chloroplast genome was sequenced by constructing large-insert fosmid clones from high molecular weight DNA as previously described in Raymond et al. [19] and as adapted in Cattolico et al. [20]. Clones were plated using 12 μg/mL chloramphenicol selection, picked using the Q-pix automated colony picker (Genetix Ltd. UK) and inoculated into 384-well glycerol stock freezing plates.

Fosmid DNA was recovered using a standard alkaline-lysis protocol, and sequenced using standard dye-termination methods and capillary electrophoresis according to ABI manufacturer’s directions using a 3730xl Genome Analyzer. Vector sequences were removed and sequences were further trimmed to remove low quality bases. Sequences were compared to a custom database consisting of published chloroplast genomes using BLASTX. Fosmids in which both end sequences had high quality matches (E value < 10^-4^) to a chloroplast gene as judged by both BLAST analyses were identified as chloroplast-derived. All fosmid end sequences are available on our web site database ( http://chloroplast.ocean.washington.edu). A total of fourteen 384-well plates were sequenced from three independent library preparations. Of those, 41 clones had end-sequences with chloroplast signatures, and these were subjected to Multiple Complete Digest (MCD) restriction analysis. Clones were analyzed by MCD analysis as previously described [19,21]. Fosmid clones were digested using *Hind*III, *Bgl*II, *Nsi*I, and *Eco*RI, subjected to electrophoretic separation on a 0.8% agarose gels, and visualized using a Typhoon 8600 Variable Mode Imager (Amersham Biosciences, Piscatawny, NJ). Automated band calling was performed using QGAP software (Quantitative Gel Analysis Program). Restriction data were analyzed using GenVal software [21] that compares DNA fingerprints and aligns end-sequence data for multiple clones, either against a reference genome or *de novo*. For this genome, three fosmids were initially sequenced that appeared to be spatially positioned to maximize genome coverage. Following sequencing and finishing (see below), two additional clones were selected for sequencing to extend the contig, but they did not complete the genome. Final finishing of the sequence was performed using experiments designed by Autofinish [22]. Each fosmid clone was finished (mis-assemblies resolved, weak regions and gaps closed) separately and then assembled in Consed. Final validation was completed by expert finishers at the University of Washington using the MCD data from the fosmids. A final gap of approximately 15 kbp gap was not covered by fosmids and was closed by sequencing PCR products that were generated using primers designed using the *N. oceanica* genome [14].

The *N. oculata* mitochondrial genome was sequenced to ~50X coverage using the Illumina Hiseq 2000 according to manufacturer’s instructions. A paired end shotgun library was prepared from total genomic DNA using the Illumina Nextera DNA sample preparation kit (Catalog #FC-121-1030) using dual indexing [23]. A total of 2.5 million 60mer reads were recovered following demultiplexing. The reads were assembled using Velvet version 1.2.03 [18]. Assembly parameters were determined empirically using a custom script which explored velvet parameter space and compared resulting assemblies against the mitochondrial genome of *N. oceanica*. Once optimum Velvet parameters were determined, all contigs greater than 1000 bp were annotated using a custom auto-annotation pipeline and mitochondrial contigs were easily identified.

**Figure 1:**
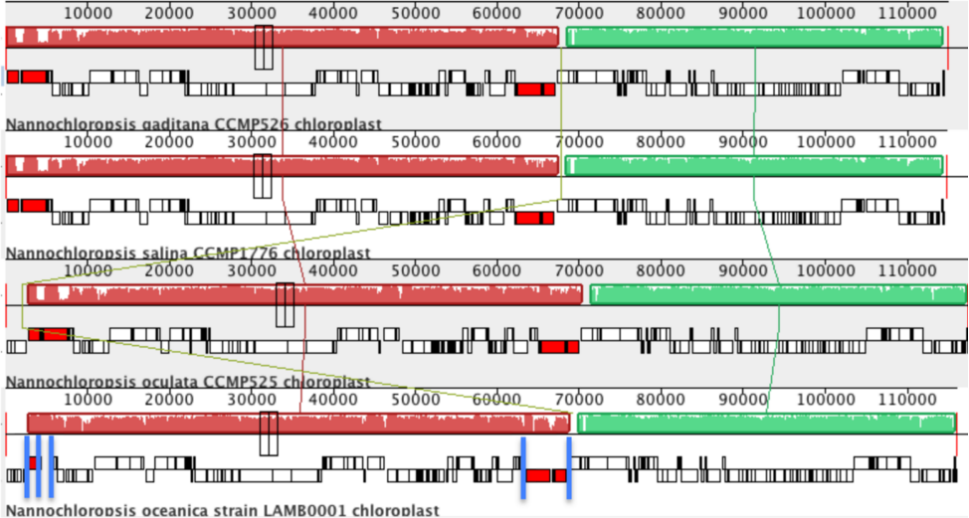
**Whole chloroplast genome alignments of *N. salina*, *N. gaditana, N. oculata*, and *N. oceanica.*** The red and green co‐‐linear blocks indicate regions of synteny and homology between the four algal species. The lines connecting the genomes indicate orthologous gene clusters. The solid blue lines indicate the locations of gaps in the *N. oceanica* genome assembly.

For *N. oceanica,* contigs containing chloroplast and mitochondria sequence from *N. oceanica LAMB0001* were retrieved from the publicly available draft assembly ( http://www.ebi.ac.uk/ena/data/view/AEUM00000000) [14] using homologous tblastx searches against the finished *N. salina* genome. *N. oceanica* contigs with a high degree of similarity (> e-50; n = 7) were scaffolded and syntenously aligned using the finished *N. oculata* and *N. salina* organellar genomes prior to annotation. The mitochondrial genome of *N. oceanica* was found be to completely assembled and the chloroplast genome was broken into seven contigs with five gaps in the assembly (see Figure 1 for locations). Due to its ‘draft’ status, some sequencing and assembly errors likely exist in the *N. oceanica* chloroplast genome. The finished chloroplast and mitochondrial genome sequences from *N. gaditana* were provided by M. Posewitz [13]. With the exception of re-orientation, the nucleotide composition of the *N. gaditana* or *N. oceanica* organellar replicons were not altered prior to annotation and analysis.

### Annotation

To enable an accurate comparative analysis, all chloroplast and mitochondrial genome sequences were syntenously aligned and reoriented upstream of the *rrn*23S and *cox*1 codons, respectively, and annotated using the same methods and employing standard genetic codes for translating open reading frames (ORFs). ORFs were initially predicted using Glimmer 3.0 [24], ribosomal RNA genes were identified with RNAmmer [25], and genes for tRNAs were identified using tRNASCAN-SE [26]. Searches for tmRNAs and the signal recognition particle RNA employed ARAGORN [27] and SRPscan [28]. Predicted gene functions were initially assigned using a BLASTP search of a custom chloroplast or mitochondrial genome database and refined manually with the aid of conserved protein motifs identified using the PFAM database [29]. Tandem repeats were found with Tandem Repeat Finder [30] using default settings. Inverted repeats were found with E-inverted from the EMBOSS package [31] using the default settings and the additional constraint that repeats had to be more than 80% similar and the length of the loop shorter than the stem. Repeats were further examines using M-fold ( http://mfold.rna.albany.edu/?q=mfold/DNA-Folding-Form) using default settings. Circular genome maps were created with OGDRAW [32]. Manual corrections to the above automated structural and functional assignments were completed on an individual gene-by-gene basis as needed.

Sequences and annotations for the chloroplast and mitochondria genomes in *Ectocarpus siliculosus*[33], *Aureococcus anophagefferens*[34], *Thalassiosira pseudonana*[1,35], *Phaeodactylum tricornutum*[1,35], and *Heterosigma akashiwo*[20] were retrieved from Genbank [ http://www.ncbi.nlm.nih.gov/pubmed].

### Genome analysis

Protein translations of all ORFs found on the *Nannochloropsis* organellar genomes were subjected to BLASTP searches against the NCBI non-redundant (NR) protein database (version 2012.10.19). Genes were considered ‘divergent’ based on the following criteria: a) e-value of the best hit was >1e-20, and b) the query and subject lengths varied by >20% or the aligned portion of the proteins had <40% similarity to the closest blast hit. Nucleotide alignments of all replicons were completed using Mauve 2.3.1 and the EMBOSS Stretcher [31] pairwise sequence alignment tool with default settings. Multi-protein sequence alignments were completed in MEGA [36] employing MUSCLE algorithms. Tertiary structure prediction for Nsk00142 (‘*clpN*’) was completed using I-Tasser [37] with default settings. Primary amino acid sequences alignments for AtpD were completed using clustalW and manually curated based on tertiary structure predictions (see Methods below). Phylogenetic trees of CbbX employed RAxML v 7.2.8 using 400 amino acid positions (excluding the C-terminal extension possessed only by *Nannochloropsis*) with rapid bootstrapping, a gamma model of rate heterogeneity and the RTREV substitution matrix.

*Ab initio* modeling [38] and comparative modeling [39] were completed using Rosetta to garner insight on structural changes encoded by the *atpD*, *atpG* and *atpA*-N terminus (first 20 amino acids of *atpA* sequence). Three and nine amino acid fragments were created from the protein database using the ROSETTA server [40]. Secondary structure predictions were made for the sequences using psipred [41]. For *ab initio* structure predictions, 16050 trajectories were run for AtpD sequence and 20400 trajectories for AtpG and the N terminus of AtpD. The models were clustered based on their RMSD and the top 20 clusters based on the total-score were visually evaluated.

For comparative modeling, the crystal structure of *Ecoli* delta-subunit (PDB code: 1abv) [42] and bovine OSCP (PDB code: 2bo5) [43] were used as templates. The sequence alignment of *N. salina* AtpD was completed using ClustalX [44] and gaps were removed manually based on secondary structure predictions for *N. salina* protein sequences using psipred and the secondary structure observed in *E coli* AtpD and bovine OSCP structures. The alignment was adjusted to place the gaps in the loop region of the template structures. 15300 trajectories were run against each template structure. The secondary structures of the extreme N and C terminal regions of all AtpD homologs not covered by crystal structures were predicted bases on consensus predictions from psipred [45] and Porter [46]. Similarly, *N. salina* AtpG was modeled against multiple structures from PDB that had close sequence homology with *N. salina* atpG identified using HHpred server [47]. A subset of structural hits were used as templates for comparative modeling (PDB codes: 3V6I, 1B9U, 1L2P, 2KHK, 2CLY, 2K88, 2KK7, 3VOU).

To determine if the *N. salina atpA* and its predicted protein structure would interact with AtpD and be in a similar orientation as the *E. coli* complex and bovine complexes, the N-terminus fragment (20 residues) from the NS-*atpA* sequence was used to estimate the structure. The top structural hit on HHpred server (PDB code: 3KKR) was used as a template for comparative modeling of NS-*atpA* N-terminus sequence. The lowest energy predicted structure was then docked in the expected pocket in the predicted AtpD structure using Rosetta docking protocol [48,49]. A total of 10200 dock trajectories were run. During the dock, the predicted AtpD structure was truncated at the C-terminus beyond the structural overlap with the template PDB (1abv in this case). Using gnuplot [50], the total predicted full-atom energy [51] of each complex was then plotted aginst the RMS deviation of each complex from the best full-atom energy complex. Another random pocket on the predicted AtpD was chosen and 30600 trajectories of docking were completed, where the AtpA-N terminus structure was randomly positioned all over the AtpD predicted structure. The total predicted full-atom energy was recorded for each docked conformation (Additional file 1: Figure S1).

### Transcript preparation, sequencing, and analysis

*Nannochloropsis salina* cells were grown as indicated above (see ‘Culturing and DNA purification’ methods). Samples (10 mL) were robotically removed on days 8, 9, and 13 during a N deprivation experiment, centrifuged at 3500 X g, flash frozen and stored at -80°C. Total RNA was extracted as follows: cells were lysed by addition of 3 mL ice cold Trizol with 1% w/v laurylsarcosine, passed three times through a cold Avestin pressure homogenizer at 36000 psi then vortexed after the addition of 750 μL of chloroform. The solution was allowed to equilibrate for 5 minutes, and then phase separated by centrifugation with phase lock gel at 13000 g for 10 min at 4°C. The aqueous phase was mixed with 100% ethanol to a final concentration of 70% and applied to an Invitrogen PureLink mRNA column (Life Technologies, Carlsbad, CA). Residual DNA was removed on column by treating with DNase.

Total RNA from each time point was separated into two aliquots. One aliquot was subjected to poly-A selection by hybridizing to poly-T coated beads using the Invitrogen Fastrack MAG mRNA Isolation Kit (Part number 45–7000; Life Technologies, Carlsbad, CA) according to the manufacturers instructions. Ribosomal RNA was removed from the second aliquot using both the Plant Leaf and Bacteria RiboZero rRNA Removal Kits (Part numbers MRZPL116 and MRZMB126; Epicentre, Madison, WI). Following these pre-treatments, both RNA aliquots were prepared for shotgun sequencing (2 X 100 base pairs) using the ScriptSeq v2 RNA-seq Library Preparation Kit (SSV21124, Epicentre, Madison, WI) and sequenced on the Illumina Hiseq 2000 platform [16], generating approximately 20 million reads per sample. Sequence reads were quality trimmed on both ends (Q > 10 sliding window), mapped to the *N. salina* chloroplast and mitochondrial genomes using Bowtie2, and RPKM values were calculated for each gene using Artemis [52]. Resultant transcript expression profiles (.bam files) were visualized via Artemis and/or IGV [52,53].

## Results and discussion

### Global characteristics and interspecies comparisons

**Figure 2:**
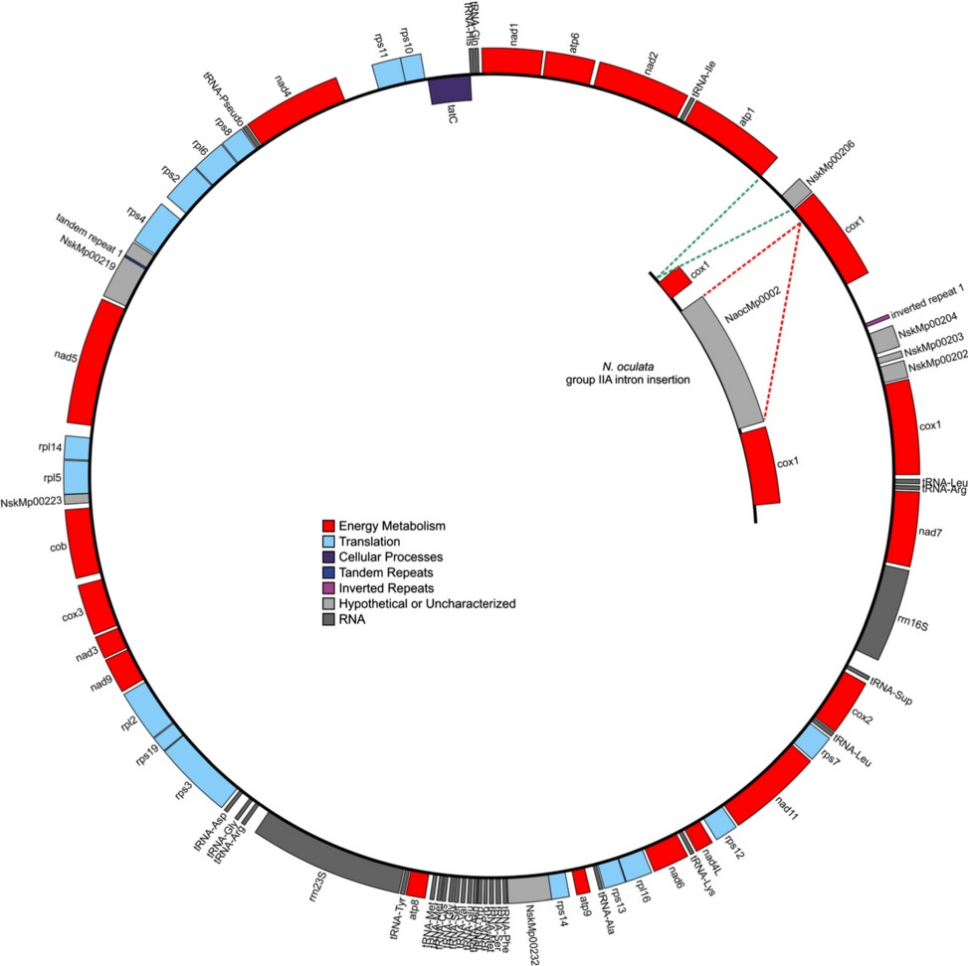
**Circular diagram of the *N. salina *mitochondrial genome.** The inset gene cluster, located on the *N. oculata* mitochondrial genome, shows genetic variation around *cox1;* insertion of the group IIA intron insertion (red dashes), and insertion of sequence in *N. salina* between cox1 and atp1 (green dashes). Genes are color-coded based on related metabolic function (see legend for categories).

The *N. salina* and *N. oculata* mitochondrial genomes are circular replicons of 41991 bp and ~41721 bp in size and contain 43 and 40 protein encoding genes, respectively (Table 1, Figure 2). They each contain single 23S and 16S rRNA genes, but lack a 5S rRNA gene. Approximately two-thirds of the tRNA coding genes found on the mitochondrial genomes are tightly clustered and are localized near the 23S rRNA (Figure 2). Differences in gene content between these mitochondrial sequences are mostly due to a) a duplication of cytochrome oxidase subunit I (*cox1*) in *N. salina*, b) variations in small reading frames with unknown function that remain unsupported by the transcriptome, and c) the unique presence of a group IIA intron that splits *cox1* in *N. oculata*. This particular group IIA intron contains the conserved 5′ and 3′-end sequences GUGCG and AC and an intron encoded protein (NaocMp0002) of the RT type with reverse transcriptase, maturase and endonuclease domains [54]. A similar group IIA intron has also been observed in the *cox1* genes of diatoms and the brown alga *Pylaiella litoralis*[55]. Several lines of evidence indicate that these introns are a result of independent insertion events [56,57]. The unique presence of a group IIA intron in *N. oculata* but not the other three *Nannochloropsis* species reinforces this hypothesis. With the exception of the *N. oculata cox1*, all other ORFs on the mitochondrial and chloroplast replicons are devoid of introns.

**Table 1:** **General characteristics of the *Nannochloropsis *** **organellar genomes**

	**Feature**	***N. salina***	***N. gaditana***^**#**^	***N. oculata***^***#***^	***N. oceanica***
		**CCMP1776**	**CCMP527**	**CCMP525**	**LAMB0001**
Chloroplast	Size (bp)	114821	114875	117463	115980^*^
	GC content	32.92	32.96	33.4	33.5
	Genes	132	132 (124)	136 (126)	136
	tRNA	28	28	29(34)	27
	rRNA	6	6	6	6
	Nucleotide identity (%)^†^	100.0	98.4	84.3	81.3
Mitochondria	Size (bp)	41992	42067	41721*	38067
	GC content	31.4	31.4	32.2	31.9
	Genes	43	43 (36)	40 (35)	41
	tRNA	27	27	26 (28)	25
	rRNA	2	2	2	2
	Intronic ORF	0	0	1	0
	Nucleotide identity (%)^†^	100.0	97.0	76.2	73.5

^†^Percent global nucleotide identity relative to *N. salina.*

^#^The quantity of genes previously reported for *N. gaditana CCMP527*[13] and *N. oculata CCMP525*[12] are shown in parentheses.

^*^Indicates the amount of assembled bases; 1 or more gaps remain in the assembly.

Seven and three novel ‘ORFans’ were annotated in *N. salina* and *N. oculata*, respectively. Two of these ORFans NskMp00219 and NskMp00232 are conserved in all four species examined and encode proteins of 323 and 231 amino acids, respectively. Based on BLASTP analysis, both of these genes do not have homologs (outside of the *Nannochloropsis*) in the NCBI non-redundant protein sequence database. Both genes appear to be transcribed as sequence reads from the transcriptome mapped to these regions. Unfortunately, tertiary structure analysis of the proteins encoded by either gene did not produce analogs with high structural similarity scores (data not shown).

**Figure 3:**
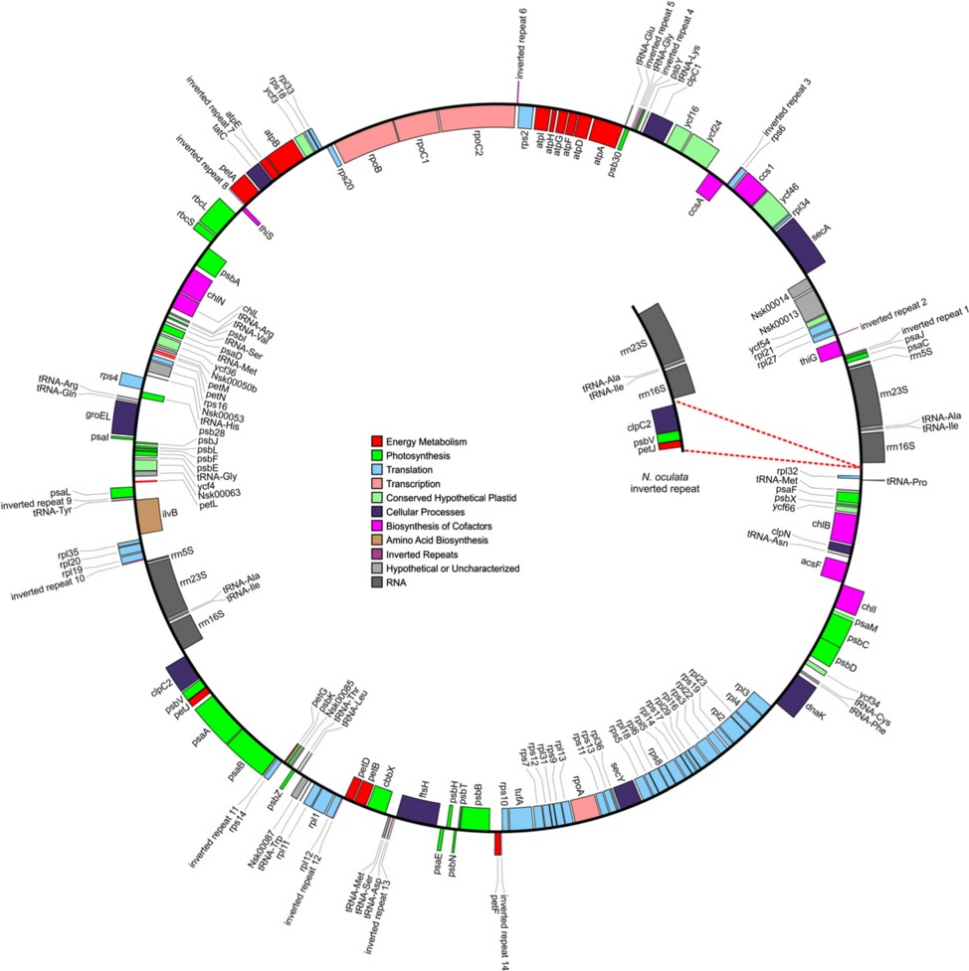
**Circular diagram of the *N*. *salina *chloroplast genome.** The inset gene cluster indicates the genomic variation of the inverted repeat in *N. oculata*. The red dashes indicates the location of the three gene deletion in *N. salina.* Genes are color-coded based on the metabolic function (see legend).

The *N. salina* and *N. oculata* chloroplast genomes were also found to be circular, containing 114821 and 117463 bp, respectively (Table 1, Figure 3). The *N. salina* chloroplast encodes 132 proteins and 28 tRNAs while the *N. oculata* chloroplast contains 136 proteins and 29 tRNAs. The gene content reported herein for the *N. oculata* orgenelles is greater than what was previously reported [12] (see Table 1 for comparisons); manual curation of automated gene predictions combined with transcriptomic and tertiary structure prediction evidence enabled the annotation of a few novel ORFs and canonical genes involved in energetics (*psaM*, *petL*, *petM*, *acpP, nad10*). Based on our initial annotation of *N. salina* and the re-annotation of *N. gaditana*, the protein and tRNA encoding content of these two organisms are identical. Similarly, the *N. oculata* and *N. oceanica* LAMB001 chloroplast genomes also appear to encode the same proteins and tRNA structures. Among these four representatives, 131 proteins were identified to be conserved among all species analyzed; *N. gaditana* and *N. salina* (pair 1) share one unique ORF (Nsk00085) not found in *N. oceanica* and *N. oculata* (pair 2). Similarly, *N. oceanica* and *N. oculata* encode two unique, small reading frames of unknown function not found in *N. salina* or *N. gaditana*. Transcription of Nsk00085 (Table 2) in *N. salina* was not detected at any of the time points sampled, therefore it remains to be determined if this reading frame and the two reading frames in pair 2 encode for functional proteins.

**Table 2:** **Pangenomes of the *Nannochloropsis *** **organelles**

**Function**	Chloroplast* (138 genes)	Mitochondria* (48 genes)
**Photosynthesis**	***psaA psaB psaC psaD psaE psaF*** *psaI****psaJ psaL psaM psb30 psbA psbB psbC psbD psbE psbF psbH*** *psbI****psbJ psbK psbL psbN psbT psbV*** *psb28****psbX psbY psbZ***	
**Cytochromes, chlorophyll, cofactor biosynthesis**	***chlI chlL chlN chlB acsF ycf54 ccsA css1 petA petB petD petF petG petJ*** *petL****petM petN *thiG** *thiS*	
**Carbon metabolism**	***rbcL rbcS cbbX ilvB acpP***	
**Energy metabolism**	***atpA atpB atpD atpE atpF atpG atpH atpI***	***atp1 apt6 apt8 atp9 cob cox1 (2) cox2 cox3 nad1 nad2 nad3*** *nad4****nad4L nad5 nad6 nad7 nad9 nad10***
**Translation**	***rpl1 rpl2 rpl3 rpl4 rpl5 rpl6 rpl11 rpl12 rpl13 rpl14 rpl16 rpl18 rpl19 rpl20 rpl21 rpl22*** *rpl23****rpl27*** *rpl29****rpl31*** *rpl32****rpl33*** *rpl34 rpl35 rpl36****rps2 rps3 rps4 rps5 rps6 rps7 rps8 rps9 rps10 rps11 rps12 rps13 rps14 rps16 rps17 rps18 rps19 rps20 tufA***	***rpl14 rpl16 rpl2 rpl5 rpl6*** *rps 2****rps3 rps4 rps7*** *rps8 rps10 rps11****rps12 rps13 rps14 rps19***
**Cellular processes**	***ftsH dnaK groEL secA secY tatC clpC1 clpC2*** (2) ***clpN sufB/ycf24 sufC/ycf16***	***tatC***
**Transcription**	***rpoA rpoB rpoC1 rpoC2***	*NaocMp0002*
**Conserved unknowns**	***ycf3 ycf4*** *ycf34****ycp36 ycf44 ycf46 ycf49 ycf66***	
**Novel hypotheticals/ORFans**^**+**^	***Nsk00013 Nsk00014 Nsk00050b Nsk00053*** *Nsk00085****Nsk00087*** *Naoc00026 Naoc00069*	***Nsk00219 Nsk00223 Nsk00232***
		***Nsk00202 Nsk00203 Nsk00204 Nsk00206*** *NaocMp0016 Naon00225 NaonMp0027 NaonMp0040*
		*NaonMp0027 NaonMp0016*

*Transcript was detected for all genes in bold.

^+^Representative genes are shown; one or more orthologs are present on the *Nannochloropsis* replicons.

All *Nannochloropsis* strains encode small inverted repeats throughout their chloroplast genomes, almost exclusively within intergenic regions (Figures 2 &3). Cruciform arrangements, formed by inverted repeats, represent alternative DNA structural elements that are known to impact a wide variety of cellular processes, including DNA replication, repair, protein association and gene expression. M-folding show these inverted repeats to have a very defined architecture wherein loop and stem sizes are highly conserved. Of the 66 inverted repeats examined, loop domains were found to be quite small. Seventy-four percent of the loop structures averaged 4.2+/- 0.8 bp in length; 23% were 7.7 +/- 1.7 bp in length while only 3% has a length of 11.5 +/- 0.7 bp. Stem size of the repeats appeared to fall into two categories. For example, those repeats servicing photosystem I genes (*psa*B, J and L) and energy conservation (*atpH,G*, E; *petA,D, F),* had an average stem length of 20.8 +/- 4.4 bp, while stem length of repeats servicing photosystem II genes (*psbH, Y, N, I, T*) had a longer length of 30.7 +/- 6.9 bp. Interestingly, *psbH*, *N*, and *I* also have the among the largest (~10 bp) loop domains. Several individual genes also have longer repeat stem structures. The *rpo*C2 and *acf*F (with stem lenths of 34 and 37 bp respectively) are good examples. Similar to bacterial gene regulation, we note that the small repeats may serve adjacent genes that are on opposite reading strands (e.g., *pet*D-*rpl*12; *pet*A-*thi*S; *ccsA*-*rps*6; *psa*J*- Thi*G; *pet*F- *rps*10). Such placement is often conserved for all four *Nannochloropsis* strains. Though these dual serving repeats are "shared" with nearest neighbors, we have found that specific genes, such as *pet*D or *rps*10, appear to be targeted, regardless of taxon for repeat embellishment (data not shown; [20]). One may speculate that the proteins encoded by these genes are seminal players in photosynthesis or transcription and may be targets for regulation.

All four *Nannochloropsis* chloroplast genomes are divided into two approximately equal coding domains by the presence of a large inverted repeat (IR). The size of this repeat has been show to be strain dependent in *Nannochloropsis*[12]. The *N. salina* and *N. gaditana* repeat encodes the 23, 16 and 5S ribosomal genes (4.9 kb). Confirming previous observations [12], *N. oculata* expands this repeat coding array to include three additional genes: *clpC2*, *psb*V and *pet*J (7.5 kb) (Figure 3 inset). Though an *N. oceanica LAMB0001* repeat structure is evident and is likely similar to IR found in the other sequenced *N. oceanica* strains [12], the publicly available genome remains incompletely assembled in both of the repeat domains (Figure 1). As more genomes are completed, the new data suggests that stramenopile IR size may generally be taxon dependent with complete loss [34] or smaller IR’s (~ 6Kb) occurring within the eustigmatophytes, pelageophytes, pinguiophytes, and xanthophytes and larger repeats (10 – 22 kb) found in the raphidophytes and bacilliariophytes ( http://chloroplast.ocean.washington.edu/home). Well-documented chloroplast genome IR size change has been extensively studied in the viridiplantae [58,59]. Outside of the *Nannochloropsis* lineage, chloroplast genome strain comparisons have only been accomplished in the stramenopiles for *Heterosigma akashiwo* (strains CCMP 452 and NIES 293; [20]), and for species comparisons made between *Thalassiosira oceanica* (CCMP1005) and *T. pseudonana* (CCMP 1335; [35]). Unlike the observation in *Nannochloropsis*[12]*,* no difference in large IR repeat size was observed either between strains or genera. Why chloroplast genomes maintain the IR domain remains undeciphered. Although the large IR structures promote the formation of molecular isomers within the chloroplast genome population [20,60] via recombination, differential function for these isomorphic forms has not been determined. However, copy correction between IR domains may contribute to genome stability- an especially important fact when one considers that an algal cell may contain hundreds of chloroplast DNA molecules [61].

Due to the high level of protein similarity and synteny encoded in the organallar genomes of these *Nannochloropsis* species, we globally aligned each organellar genome and examined the relative nucleotide similarity of each species (Table 1, Figure 1). Although the *N. oculata* mitochondrial genome is closer in size to *N. salina* than *N. oceanica*, the nucleotide similarity of *N. oculata* is most similar to *N. oceanica* (*N. oculata* vs. *N. salina*; 76.2%, *N. oculata* vs. *N. oceanica*; 87.7%). Similarly, the entire *N. salina* and *N. gaditana* mitochondrial genomes share a 97% nucleotide identity and a 100% conservation of gene synteny. With respect to the chloroplast genomes, the *N. salina* and *N. gaditana* replicons only differ by 75 bp, are 98.4% identical at the nucleotide level, and contain an identical inventory of open reading frames. In contrast, the *N. oculata* chloroplast sequence is only 84.3% identical to *N. salina* but is 92.4% identical at the nucleotide level to *N. oceanica*. Taken together, these data indicate that the *N. salina* and *N. gaditana* replicons are more similar to each other than they are to the organellar genomes found in *N. oculata* and *N. oceanica* which is consistent with previously known evolutionary relationships [2] and a recent phylogenomic study of these organelles [12].

Because of the high degree of nucleotide similarity in the organellar genomes of *N. salina* and *N. gaditana*, a re-assessment of the phylogentic placement of *N. gaditana* is warranted. To our knowledge, no dogma has been established to phylogenetically classify single cell eukaryotes strictly based on the degree of nucleotide variation in highly conserved genes. As a general rule in bacteria, if two different bacterial isolates contain 16 s rDNA genes that are ≥ 97% similar, they are classified as the same species. The chloroplast ribosomal RNAs in *N. salina* and *N. gaditana* only differ by 7 nucleotides (99.76% identical). As stated above, we observed ≥ 97% nucleotide similarity across the entire mitochondrial and chloroplast replicons of *N. salina CCMP1776* and *N. gaditana CCMP526.* If NADH dehydrogenase subunit 5 (*nad5*) is used as a strain discriminator, a higher resolution among organisms can be achieved (Black and Cattolico, unpublished). Little difference in nucleotide sequence diversity in *nad5* is observed when either *N. salina* and *N. gaditana* (1.6% difference) or *N. oculata* and *N. oceanica* (5.8% difference) are compared. In contrast, comparisons between *N. salina* and *N. oceanica* or *N. gaditana* and *N. oculata nad5* indicate 14.6% and 15.5% sequence variation, respectively. These data provide further support that *N. salina* and *N. gaditana* are closely related. In conclusion, the identical gene synteny and high degree of nucleotide identities suggest that *N. gaditana* could be reclassified as a strain of *N. salina* (i.e. "*Nannochloropsis salina* strain gaditana"). The availability and consequent comparative analysis of the nuclear genomes from both isolates will undoubtedly provide clarifying evidence to support this proposition.

### Intergenus comparisons

To gain further insight into the unique features conserved within the genus *Nannochloropsis*, we compared the gene content of the *Nannochloropsis* organellar pangenomes (Table 2) to other representative sequences found in the same phyla (*Thalassiosira pseudonana* (Coscinodiscophyceae), *Phaeodactylum tricornutum* (Bacilliariophyceae), *Ectocarpus siliculosus* (Phaeophyceae)*, Aureococcus anophagefferens* (Pelagophyceae), and *Heterosigma akashiwo* (Raphidophyceae) [20]. Within this set of stramenopiles, the global gene inventory of *Nannochloropsis* is most similar to *H. akashiwo* (data not shown). Unlike *A. anophagefferens, P. tricornutum, T. thalassiosira,* and *H. akashiwo,* the *Nannochloropsis* and *Ectocarpus* chloroplast genomes both contain *chlB*, *chlN*, *chlL* (light independent protochlorophyllide reduction), the *acsF*/*chl27* (Mg-protoporphyrin IX monomethyl ester cyclase) gene as well as *ycf54* (demonstrated to play a critical role in AcsF synthesis/maturation or in the process of cyclase assembly [62]). This gene assemblage suggests that these stramenopile genera (*Nannochloropsis*, *Ectocarpus*, and others with the same gene complement) may share similar mechanisms of chlorophyll biosynthesis*.* The *Nannochloropsis* chloroplasts have also maintained single copies of *petJ*, *ycf49*, *ycf36*, genes more typically conserved in cyanobacteria, rhodophytes and some stramenopiles (e.g., xanthophytes and raphidophytes) but are usually found to be transferred to the nucleus in the bacilliariophytes. Additionally, all four *Nannochloropsis* mitochondria encode *atp1,* a subunit of the F1F0 ATP synthase. This gene is absent in all other stramenopile mitochondrial genomes sequenced to date.

In all four *Nannochloropsis* mitochondria, the gene which encodes for subunit ‘G’ of the NADH dehydrogensase, *nad11,* is shorter than what is canonically known, containing only the molybdopterin cofactor binding domain but lacking the NADH iron-sulfur (Fe-S) binding region. A gene which encodes a very similar Fe-S binding domain was located in the drafted *N. salina* nuclear genome, indicating that this portion of the protein is now encoded by the nuclear genome. In *P. littoralis,* the opposite transfer occurred as only the Fe-S domain is present in the mitochondrial genome and the molybdopterin binding domain is encoded in the nucleus [63]. The fact that the *P. tricornutum nad11* is split into two parts corresponding to these two domains in *N. salina* and *P. littoralis*, but that the domains still reside on the mitochondrial genome [1,35] suggests that this protein is a vulnerable target for nuclear transfer.

As in all other chloroplasts [64], many structural subunits of Photosystems (PS) I and II are conserved in the *Nannochloropsis* chloroplast genomes. Nevertheless, the PS subunits that have been lost from the chloroplast (through migration or deletion) follow previous deletion patterns observed in several stramenopile and rhodophytic representatives. Similar to what has been observed in stramenopiles [33], the PSI subunit genes *psaG, psaH*, *psaK*, *psaN*, *psaO*, *psaP*, *psaX* and the PSII subunits *psbM*, *psbP*, *psbQ*, *psbR*, *psbS* have been removed from the chloroplast genomes of *Nannochloropsis.* As seen in rhodophytic algae [64], the genes encoding PsbO, PsbU, and Psb27 are also absent in the *Nannochloropsis* genome.

Carbon dioxide fixation in *Nannochloropsis* is mediated by a ‘red-type’ Form 1 ribulose-1,5-bisphosphate carboxylase-oxygenase (RuBisCO) [65], shown to have a high affinity for CO_2_ yet a low specificity factor due to poor discrimination between O_2_ and CO_2_[66]. Single copies of *rbcL* and *rbcS* are found on each of the chloroplast genomes but a gene which encodes for the transcriptional regulator, *rbc*R was not identified in *N. salina* and is consistent with previous observations in *Nannochloropsis*[12,13] and in some of the other stramenopiles ( [67]; unpublished results). In viridiplanta and some algae, RuBisCO is post-translationally regulated via nitrosylation of conserved cysteine residues in RbcL (Cys 460 and Cys181 in *G. suphuraria*), resulting in inactivation of the enzyme at the active site [68-70]. Interestingly, the *Nannochloropsis* RbcL does contain a cysteine at position 460 but does not encode a cysteine near the active site at position 181, which suggests that this type of post-translational control may not be functioning in *Nannochloropsis*.

The *Nannochloropsis* chloroplast pangenome contains an ortholog of the large subunit of an acetohydroxyacid synthase (i.e. *ilvB*, Nsk0066), which is the only known enzyme to catalyze the first step in biosynthesis of branched chain amino acids; valine, leucine and isoleucine. Surprisingly, the accompanying ‘small subunit’ regulator, *ilvH/N*, required for negative feedback regulation and optimum activity [71-74], appears to have been uniquely lost from this genera as an ortholog of *ilvH* was not identified in any of the sequenced *Nannochloropsis* genomes (nuclear, mitochondria, or plastids). With respect to all publicly available stramenopile choloroplast genomes, either a.) *ilvB* and *ilvH* have both been maintained (i.e.,. *H. akashiwo*, *E. siliculosus*, *A. anophagefferens*) or b.) both subunits have been transferred to the nuclear genome (i.e., *T. psuedonana* and *P. tricornutum*). Searching broadly across photosynthetic organisms in other eukaryotic phyla, we could not identify another instance where *ilvH* or *ilvB* had been lost from any chloroplast genome independent of its partner gene, which is a striking occurance considering a recent review indicated all known acetohydroxyacid synthases contain both subunits [71]. Therefore, the absence of *ilvH* suggests that *Nannochloropsis* has either lost its ability to negatively regulate IlvB or has evolved a novel regulator.

### Divergent genes

Despite the fact that many genes were found to be conserved among the different classes of stramenopiles, several *Nannochloropsis* genes were identified that are highly divergent from any previously identified orthologs (Table 3). With respect to the mitochondrial genomes, significant drift in the primary amino acid sequences of the ATP synthase subunit 8 (*atp8*) and seven ribosomal subunits were discovered. Within the algae, *atp8* has previously been observed to vary significantly in length [55]. With regard to divergent chloroplast genes, the aligned portions of *ycf4*, *ycf49*, and *ycf34* are only 29%, 35%, and 28.4% similar to the closest homologs found in a random array of photosynthetic organisms. The chloroplast ORF Nsk00053, is also highly divergent at the primary amino acid level, but based on tertiary structure prediction, may share some structural similiarities with peroxidases (data not shown). As described in detail below, the RuBisCO activase and several subunits of the ATP synthase were also highly divergent from the nearest functional homolog (Table 3) and novel evolutionary modifications to vital protein homeostasis components were identified:

**Table 3:** **Highly divergent genes on the *Nannochloropsis *** **organellar genomes**

Gene^*^	**(Putative) function**	**Closest homolog**	**Query length**	**Subject length**	**Alignment length**	**Identity**^**#**^	**E-value**
Nsk00019	rps6; 30S ribosomal protein S6	30S ribosomal protein S6 [Thalassiosira pseudonana];|YP_874616.1|	106	103	96	36.5	2.00E-11
Nsk00027	atpD; Atp synthase delta subunit	Hypothetical protein MldDRAFT_4321 [delta proteobacterium MLMS-1]; |ZP_01290127.1|	232	331	162	24.7	0.02
Nsk00028	atpF; ATP synthase b subunit	CF0 subunit I of ATP synthase [Oltmannsiellopsis viridis]; |YP_635887.1|	155	183	106	33	5.00E-06
Nsk00029	atpG; ATP synthase b’ subunit	ATP synthase CF0 subunit II [Vaucheria litorea];|YP_002327468.1|	160	154	145	29.7	7.00E-11
Nsk00053	Hypothetical; putative peroxidase	Hypothetical protein tlr1577 [Thermosynechococcus elongatus BP-1];|NP_682367.1|	195	99	54	35.2	0.072
Nsk00055	psb28; photosystem II protein (ycf79)	Photosystem II protein W [Guillardia theta]; |NP_050669.1|	113	116	94	30.9	2.00E-04
Nsk00062	ycf4; photosystem I assembly protein	Photosystem I assembly protein Ycf4 [Coccomyxa subellipsoidea C-169]; |YP_004222004.1|	195	189	155	29	4.00E-12
Nsk00063	ycf49; DUF2499	Unknown DUF2499 [Picea sitchensis]; |ABK25760.1|	97	216	88	35.2	5.00E-11
Nsk00087	Unknown; ORFan	Hypothetical protein SPPN_02855 [Streptococcus pseudopneumoniae IS7493	117	282	85	25.9	0.81
Nsk00113	rpoA; RNA polymerase alpha chain	RNA polymerase alpha subunit [Cryptomonas paramecium]; |YP_003359271.1|	447	310	195	34.9	4.00E-14
Nsk00135	ycf34	Chloroplast protein Ycf34 [Gloeobacter violaceus PCC 7421]; |NP_927340.1|	86	80	81	28.4	0.27
Nsk00142	clpN	ATP-dependent Clp protease ATP-binding subunit ClpA [Desulfobulbus propionicus DSM 2032; |YP_004196194.1|	149	756	96	29.2	2.4
Nsk00202	Unknown; ORFan	Predicted protein with ABC transporter signatures [Fibroporia radiculosa]; |CCM01526.1|	93	613	54	38.9	1.3
Nsk00204	Unknown; ORFan	Hyp. periplasmic binding protein MARHY3762 [Marinobacter hydrocarbonoclasticus ATCC 49840]; |YP_005431639.1|	119	404	50	46	1.9
Nsk00206	Unknown; ORFan	Coiled-coil domain-containing protein 141 [Nomascus leucogenys]; |XP_003253834.1|	99	1530	59	37.3	5.5
Nsk00212	rps10; 30S ribosomal protein S10	30S ribosomal protein S10 [Spirochaeta smaragdinae DSM 11293]; |YP_003802682.1|	112	102	84	36.9	5.00E-05
Nsk00213	rps11; 30S ribosomal protein S11	30S ribosomal protein S11, partial [uncultured bacterium]; |EKD46317.1|	156	140	110	39.1	2.00E-18
Nsk00217	rps2; 30S ribosomal protein S2	Hypothetical protein [Batrachochytrium dendrobatidis JAM81];|EGF78568.1|	212	195	169	29	5.00E-16
Nsk00218	rps4: 30S ribosomal protein S4	Ribosomal protein S4 [Synedra acus]; |YP_003359457.1|	241	246	176	33.5	4.00E-09
Nsk00219	Unknown; ORFan	Hypothetical protein [Trichomonas vaginalis G3]; |XP_001579587.1|	323	744	118	28	1.8
Nsk00222	rpl5; 50S ribosomal protein L5	Ribosomal protein L5 [Thalassiosira pseudonana]; |YP_316605.1|	179	178	176	34.7	8.00E-19
Nsk00231	atp8; ATP synthase F0 subunit 8	ATP synthase F0 subunit 8 [Fucus vesiculosus; |YP_448633.1|	105	53	60	51.7	2.00E-07
Nsk00232	Unknown; ORFan	fmhA protein [Staphylococcus saprophyticus ATCC 15305]; |YP_300577.1|	231	410	156	23.08	6.7
Nsk00235	rps13; 30S ribosomal protein S13	NADH dehydrogenase s9- S13 fusion protein [endosymbiont of Durinskia baltica] |gb|AEP20701.1|	118	310	117	41.9	8.00E-18
Nsk00013, Nsk0014, Nsk00150, Nsk00085, Nsk00203, Nsk00223	Unknown; ORFans	No homologs	-	-	-	-	-

*Genes with locus tags that have a numerical value of <200 and > 200 are located on the chloroplast and mitochondrial genomes, respectively. Nsk00013, Nsk0014, Nsk00150, Nsk00085, Nsk00203, Nsk00223 were also identified as highly divergent with no BLASTP hit in the NR database.

^#^Identity of the aligned amino acids.

#### RuBisCO activase

**Figure 4:**
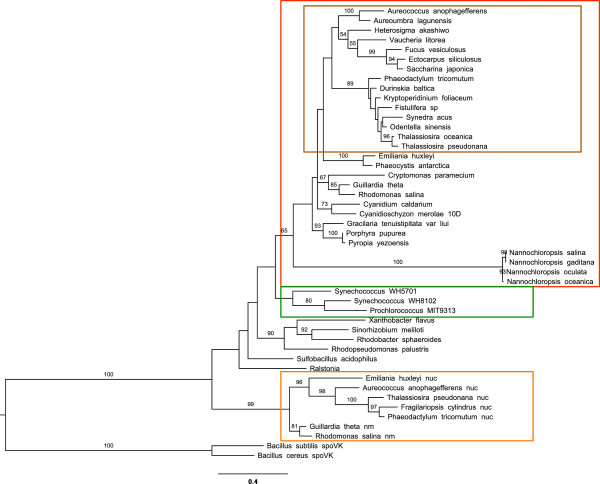
**CbbX phylogenetic tree.** Bootstrap values higher than 50% are indicated at the nodes. The scale bar represents 0.4 mutations per site. Branch lengths are drawn to scale. Cyanobacterial CbbX sequences are boxed in green. CbbX sequences from red algae or secondary endosymbiotic events with red algae are boxed in red. CbbX sequences from all Stramenopiles (except *Nannochloropsis*) are boxed in brown. CbbX sequences encoded in the nucleus (nuc) or nucleomorph (nm) are boxed in orange. The *Bacillus subtilis* sporulation factor SpoVK is used as the outgroup.

A divergent homolog of the gene which encodes a RuBisCO activase (*cbb*X/*cfx*Q) was identified in all four *Nannochloropsis* chloroplast genomes. Recently, the protein product of *cbbX* was shown to function as a red-type RuBisCO activase in the proteobacterium *Rhodobacter spheoroides*[65], a modern bacterial relative of the proteobacteria from which the algal red-lineage obtained the RuBisCO operon and most likely the *cbbX* gene by lateral gene transfer [75]. In this organism, CbbX activates RuBisCO by pulling on a carboxy-terminal extension of RbcL (not present in chlorophytes) into the central pore of the CbbX hexamer, thereby changing the conformation of RuBisCO and releasing inhibitory RuBP [65]. The CbbX in *N. salina* and *R. sphaeroides* are only 43% identical at the protein level yet the vast majority of residues shown to be required for normal activase function (atpase activity, binding of RuBP, and hexameric structural stability) in *R. sphaeroides*[65] are highly conserved in the *Nannochloropsis cbbX*. The *Nannochloropsis* CbbX sequence is quite divergent from the CbbX of other stramenopiles (Figure 4), but is not specifically closely related to bacterial or nuclear-encoded sequences, suggesting that rapid evolutionary divergence rather than lateral transfer is responsible for the long branch lengths. Furthermore, the *Nannochloropsis* CbbX has a ~45 amino acid carboxy terminal extension relative to *R. sphaeroides* and all other stramenopile and red lineage plastid encoded cbbX genes; the functional role of this extension is unclear. The amino acids which make up the conserved motif in the pore loop of the assembled CbbX hexamer, Y(I/V)G, have been slightly modified in *Nannochloropsis* to ‘FVG’. With respect to the large subunit of RuBisCO, RbcL, the *Nannochloropsis* homolog has maintained a carboxy-terminal extension (Additional file 2: Figure S2) but the amino acid sequence has diverged from that found in proteobacteria and rhodophytes and has also been shortened by one residue. The deletion of one amino acid from the C-terminus is so far unique to *Nannochloropsis* among the stramenopiles. Deletion of the terminal residue from CbbX in *R. sphaeroides* did not significantly alter atpase or activase activity [65], although further research will be required to assess if the other amino acid changes in CbbX and the loss of the nitrosylation site in RbcL (described above) can help explain the observed biochemical activity of RuBisCO in *Nannochloropsis*.

#### ATP synthase gene cluster

As indicated previously, many of the *Nannochloropsis* ATP synthase genes have diverged significantly from all other F_1_F_0_ type ATP synthases across the tree of life. Like most other algae, the *Nannochloropsis* chloroplast genomes encode an F_1_F_0_ type ATP synthase, a multimeric complex that catalyzes the synthesis of ATP from energy conserved through photosynthesis [76,77]. The F_1_ complex (stator), which houses the catalytic site, is encoded by the alpha, beta, and delta subunits (AtpA, AtpB, AtpD) and interacts structurally with the central (gamma subunit) and peripheral (b/b’ subunits; AtpF/G) stalks to connect and stabilize the F1 to the membrane bound F0 complex (rotor). All previously published annotations of the *Nannochloropsis* chloroplast genomes genomes indicated that *atp*D was not present on the replicon [12,13,78], and analysis of the *Nannochloropsis oceanica* CCMP 1779 genome indicated that *atpD* was possibly located in the nuclear genome [78]. Although *atp*D is often tandemly transferred to the nuclear genome with *atpG* in other algae [79], a close homolog of *atpD* could not be identified in the drafted nuclear genomes of *N. gaditana, N. salina,* or either *N. oceanica* genome*.* Because AtpD has been shown to be essential for function of the ATP synthase complex in yeast [80] and bacteria [81,82] and similarly, loss of *atpD* expression in *Arabidopsis* disabled photoautotrophic growth [83], we hypothesized that a functional replacement (or a highly diverged ortholog) must be present on the chloroplast or nuclear genome.

**Figure 5:**
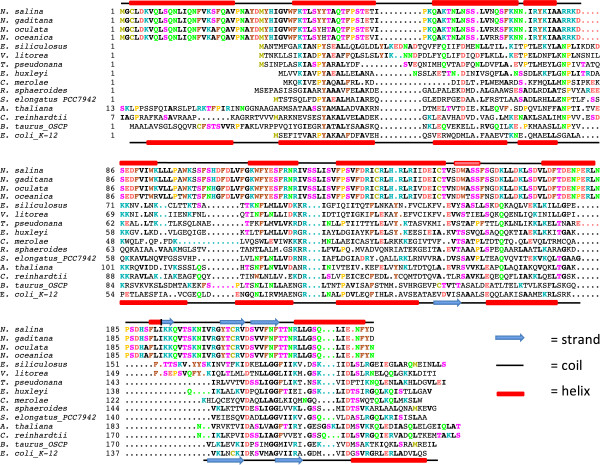
**Primary and secondary structures of AtpD variants.** The secondary structures above the first sequence indicate the predicted secondary structure of the AtpD found in *Nannochloropsis*. The secondary structures depicted below the last sequence indicate the approximate location of the consensus secondary structures of the *E. coli* ATP synthase delta subunit and the bovine OSCP derived from the predictions made by PSIPRED and Porter.

**Figure 6:**
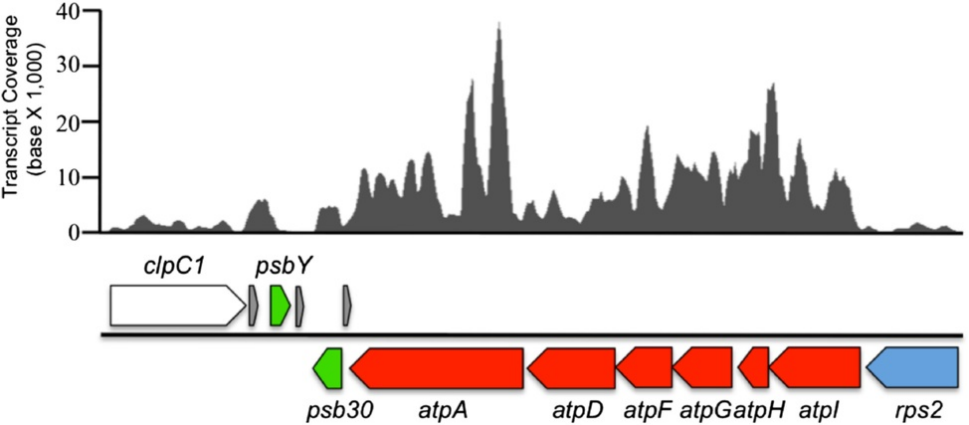
**Transcript profiles of the ATP synthase genes in *N. salina*.** The top panel indicates the coverage of transcript reads mapped to the given region of *N. salina* chloroplast genome. The bottom panel indicates the locations of the coding regions of the ATP synthase genes (red) and neighboring genes (blue, green, white). The arrowed blocks in gray indicate the location of t‒RNAs (from 5′ to 3′; tRNA‒Lys, tRNA‒Gly, tRNA‒Glu).

In the canonical location of *atp*D within the ATP synthase operon, an unannotated ORF (Nsk00027) was found to be conserved across all publicly available *Nannochloropsis* chloroplast genomes. The translated protein sequence from this ORF aligns poorly with canonical AtpD protein sequences from viridiplanta, stramenopiles and rhodophytes (Figure 5). To determine whether this ORF was a functional replacement of the canonical *atp*D, transcriptome sequences recovered during a nitrogen-limited growth study (see Methods for details) were mapped to the chloroplast genome. The entire ORF was co-transcribed with the other ATP synthase genes at every time point examined (Figure 6).

Given the extreme level of divergence in the *atpD* nucleotide and amino acid translation, we also investigated changes in the main ATP synthase subunits known to interact with the delta subunit: AtpA and AtpG. Overall, the amino acid sequence alignments of the *Nannochloropsis* AtpA display a high level of conservation with other AtpA proteins (Additional file 3: Figure S3) yet, the N-terminal amino acids, which have been shown to interact with AtpD [43,84,85] have diverged. Similarly, the N-terminus of the b’ subunit (*atp*G), which anchors the protein in the chloroplast membrane, is conserved although the C-terminal end, which interacts with AtpD, aligns poorly with canonical AtpG sequences (Additional file 4: Figure S4).

### Structure prediction and comparative modeling of the ATP synthase subunits

**Figure 7:**
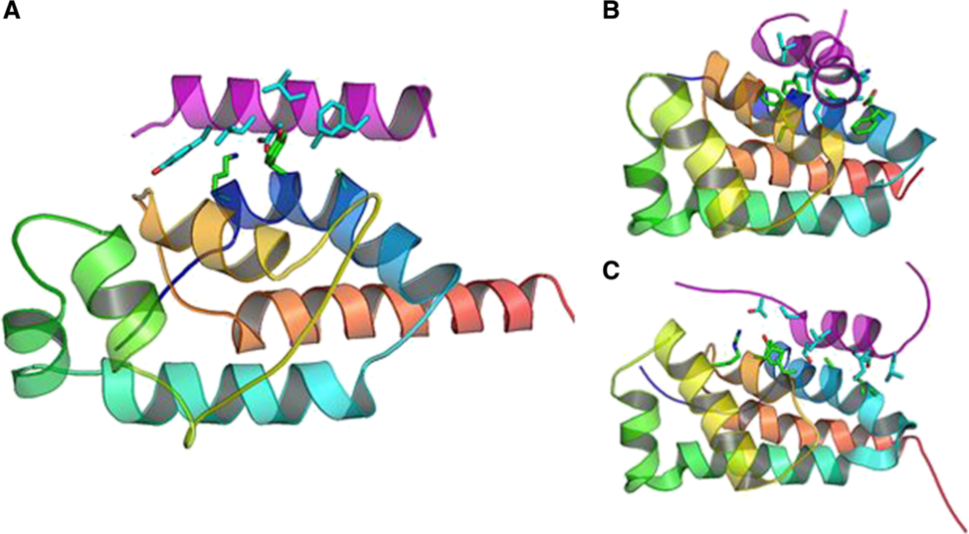
**Structural models of ATP synthase subunits.** Intermolecular interaction between N-terminal sequence of AtpA and homologs of AtpD: Minimum energy docked conformation of predicted *N. salina* AtpA-N terminus and *N. salina* AtpD (residues 31-154) **(A)**, NMR structure of *E. coli* AtpA N-terminal and *δ*-subunit (PBD code: 2A7U) **(B)**, NMR structure of Bovine AtpA N-terminal and OSCP subunit (PDB code:2JMX) **(C)**.

*Ab initio* protein secondary structures encoded by the *N. salina atpD* (Ns-AtpD, *aptG* (Ns-AtpG) and *atpA* (Ns-AtpA) N terminus (first 20 amino acids of NS-AtpA sequence) were predicted (Additional file 5: Figure S5). The Ns-AtpD subunit is largely helical and a small portion of the C-terminus shows a propensity to form β-strands. These features are very consistent with the secondary structure observed in the low resolution crystal structure of the ortholog in bovine and *E. coli* ATP synthase [84] (Figure 7B & C, Additional file 6: Figure S7A).

To gain insight into the tertiary structures of Ns-AtpD, Ns-AtpG and Ns-AtpA-N terminus, molecular docking and comparative modeling using known structures in the PDB database were conducted. Because the Ns-AtpD amino acid sequence was very divergent from any known structures, only low scoring homologies were observed on the HHpred server. Therefore, known structures of homologs from *E. coli* (PDB code: 1abv) and bovine (PDB code: 2bo5) AtpDs were used for comparative modeling. The predicted models for Ns-AtpD consistently acquired similar folds as those observed in the *E. coli* and bovine homologs (Figure 6A-C). With respect to Ns-AtpG, comparative modeling identified 43 PDB structures with some degree of sequence homology. The selected model for Ns-AtpG (Additional file 7: Figure S6F, Additional file 8) is based on the template structure 2K88 (Additional file 6: Figure S7B) and is similar to canonical AtpG structures with a long helix with breaks only towards the ends. This model strongly suggests that NS-AtpG sequence is an ortholog of the b’ subunit of the ATP synthase. The Ns-AtpA-N terminus model was arbitrarily placed in the proximity of Ns-AtpD model between the helices which correspond to the helices that interact with AtpA sequence in *E. coli* and bovine complexes (Figure 7B & C, Additional file 9). Rigorous random local docking accompanied by complete randomization of the Ns-AtpA N terminus generated a top scoring conformation similar to known AtpA-AtpD interactions. Another set of 30600 trajectories of local docking but from a different starting point failed to produce a Ns-AtpD/Ns-AtpA-N terminal conformation with a better total energy of the complex.

Approaching this analysis critically, *ab initio* modeling minimizes the structural energy by producing maximum interactions, which results in compacted AtpD, Atp-A, and AtpG structures (Additional file 7: Figure S6A-C) that deviate from the structures of the known homologs. In an ATP synthase structure, there are multiple subunits and each interact with one another to provide a stable complex [84]. Thus, in the absence of intermolecular domain-domain interactions, the predicted top scoring *ab initio* tertiary structures are likely artificial. Nevertheless, given that the *ab initio* secondary structure predictions and the the comparative modeling of tertiary structure were remarkably similar to known homologs, and the fact that the modified *Nannochloropsis atpD* is transcribed and present in the same canonical location, strongly suggests that Nsk00027 encodes a functional AtpD.

#### Clp protease complex

ATP dependent chaperone-protease complexes (Clp) play a critical role in protein homeostasis in both photosynthetic and non-photosynthetic bacteria and eukaryotes. All extant Clp complexes contain two functional elements: a chaperone protein and a proteolytic core. The bacterial chaperones (or ‘unfoldases’) ClpA, ClpC, and ClpX are members of the Clp/Hsp100 family of AAA + proteins, which function to recognize, unfold, and deliver polypeptides to the ClpP protease for degradation. Functional ClpCP complexes require an adaptor, MecA, to recruit specific protein substrates to ClpC [86]. Similarly, the related but distinct ClpAP complex utilizes an adaptor, ClpS, to recruit N-end rule substrates to ClpA [87,88].

Intriguingly, a homolog for a MecA adaptor gene (as indicated above, MecA interacts with ClpC chaperones) could not be identified on any *N. salina* replicon although two genes containing the conserved domains for ClpS (which normally interact with ClpA type chaperones) were found in the drafted nuclear genome assembly. With respect to ClpP, the *Nannochloropsis* chloroplast and mitochondria pangenomes are likewise devoid of genes which encode the ClpP protease, yet the drafted nuclear genome was found to contain five separate ORFs with putative ClpP protease domains (Additional file 10: Table S1).

**Figure 8:**
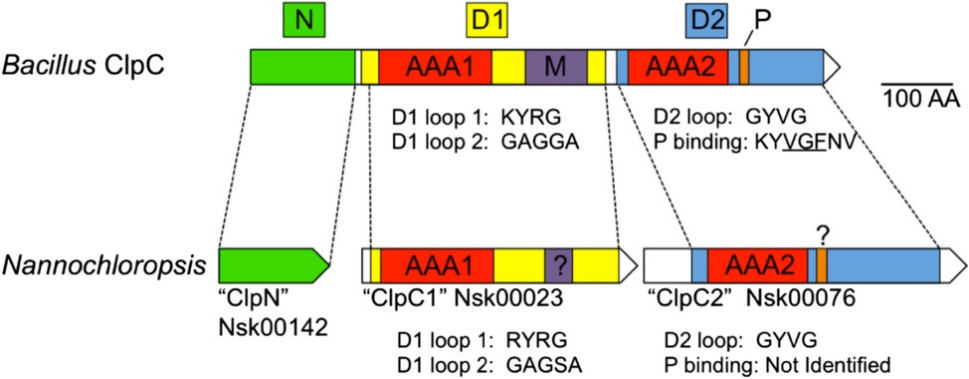
**Divergence of the *Nannochloropsis *chloroplast Clp orthologs.** ClpC contains several conserved domains: an N-domain (green), a D1-domain (yellow), a middle domain (M, purple), and a D2-domain (blue). The D1 and D2 domains each contain an AAA module (red). The D-2 domain in *Bacillus* contains a conserved ClpP-binding loop (P, orange). Homologous structural and functional features identified between bacterial ClpC and translated *Nannochloroposis* Clp orthologs are color matched. The question marks indicate that the M-domain and ClpC-binding regions were not clearly identified.

With respect to ClpC, genomic components of the *Nannochloropsis* chaperone may have evolved into novel independent components (Figure 8). The *Nannochloropsis* mitochondrial genomes are devoid of Clp homologs, yet all *Nannochloropsis* chloroplasts contain two or three (*clpC2* is duplicated as a part of the IR in some species [12]) gene homologs of *clp*C, respectively. Canonical ClpC genes encode for proteins of 800+ residues which contain several conserved domains, a Clp ‘N-domain’ which binds the adaptor, and two separate AAA domains, the first (D1) promoting ATP-induced hexamerization and the second (D2) functions to hydrolyze ATP after assembly. Structurally, the N-, D1- and D2- domains are ‘stacked’ on top of each other and collectively form the central pore for delivery of proteins to the ClpP protease and binding pockets for ATP [89]. With respect to *N. salina*, each ‘clpC-like’ gene encodes for proteins of 384 (*clpC1*; Nsk00023) and 449 (*clpC2*; Nsk00076) residues, respectively. The *N. salina clpC1* and *clpC2* each contain a single AAA domain but are not orthologous. The amino acid residues which form pore loops 1 and 2 in the D1 domain are conserved in the translated product of *clpC1* yet the M-domain that helps bind the MecA adaptor was not identifiable. In constrast, *clpC2*, when translated, encodes residues indicative of a D2 pore loop (AA residues 190–209), including the GYVG motif, thought to be required for substrate unfolding and translocation into the protease yet, the ClpP-binding loop present in bacterial ClpC D2-domains [90] has been deleted or has diverged significantly in the *Nannochloropsis* ClpC2. Because neither *clpC1* or *clpC2* appear to encode a Clp N-domain, we searched for other ORFs on the chloroplast that may have structural similarties to the N terminus of canonical ClpA or ClpC unfoldases. Indeed, the translated product of a small ORF, Nsk00142 (based on BLASTP analysis) had very weak homology to a "clpA-like" protein. Results from the protein structure prediction tool, I-TASSER, indicated that Nsk00142 potentially encodes a structural analog of canonical Clp N-domains within the Hsp100/Clp family (data not shown).

To our knowledge, this is the first observation of a complete disassembly of individual ClpC domains into separate reading frames in any organism. Although it is currently unknown if these new chloroplast encoded ‘subunits’ still function collectively with the other nuclear encoded Clp components to create an active protease complex, it is still interesting to speculate on how the Clp homologs present in *Nannochloropsis* may interact and/or how these modifications change the function of the proteins. If we first envisage a traditionally functioning ClpCP, *Nannochloropsis* could have adapted to utilize ClpS as an adaptor for ClpC due to the absence of MecA (and other known bacterial) orthologs. This suggestion is not without precedent since interactions between MecA and the N-domain of ClpC were shown to resemble those of ClpS and the N-domain of ClpA [86], and in the cyanobacteria *Synechococcus elongatus* (which is also devoid of a MecA), ClpC was shown to interact directly with ClpS *in vitro*[91]. Furthermore, because the N-domain is thought to partially mask the pore, separation of the ‘clp-N’ domain could increase the degradation efficiency by other mechanisms. For example, in the ClpAP system, SsrA-tagged substrates compete with ClpS recognized proteins for delivery to the unfoldase [92]. If an SsrA-dependent system was present in *Nannochloropsis*, physical separation of the N domain would enable unhindered access to the active site and freely enable ClpS-independent proteins to be degraded.

Because the *Nannochloropsis* ClpC2 does not contain an obvious ClpP binding loop and the M-domain in D1 is either modified (or missing), we must also consider the alternative that a canonical ClpCP complex does not function in *Nannochloropsis* and that the single domain ClpC proteins have developed specialized functions and may act independent of adaptors. The *Nannochloropsis* ClpC2 protein has a conserved D2 loop, and if hexamerized, may continue to function as an unfoldase and promiscuously deliver substrates to the ClpP protease. Furthermore, as has been shown for several bacterial ClpC orthologs [91,93,94], *clpC1* could compliment the activity of *clpC2* (acting dependent or independent of adaptors) by stabilizing and preventing aggregation of newly synthesized, unfolded proteins; a function that is essential to effectively assemble (or dispose of) large multimeric complexes in the chloroplast. Clearly, further experimentation is required to determine if these or other scenarios explain the functional role of these novel Clp orthologs.

## Conclusions

A pangenomic comparison of the *Nannochloropsis* with other stramenopiles revealed an extreme divergence in several key metabolic genes/systems: amino acid synthesis, carbon fixation, energy conservation, and protein homeostasis. These observations and further discovery of (as yet) currently unidentified genetic and structural modifications to critical cellular components will explain the unique physiological properties found in the genus *Nannochloropsis.* It is worthy to note that the high degree of divergence in the amino acid sequences of many *Nannochloropsis* proteins led to false annotations. Thus, implementation of tertiary structure prediction during annotation will be crucial to improve de novo gene calls in all newly sequenced organisms. Finally, the extraordinary similarity of the *N. salina* and *N. gaditana* organellar genomes suggests that these two isolates should be reclassified as different strains of the same species.

### Availability of supporting data

The protein models (.pdb files) for AtpG and AtpD supporting the results of this article are available as Additional files 8 and 9, respectively. The genome assemblies and annotation data sets for each organelle are available in the GenBank repository; *N. salina* CCMP1776 organelles; accession numbers KJ410685 and KJ410689; *N. oculata* CCMP525 organelles; accession numbers KJ410684 and KJ410688; *N. gaditana* CCMP526 organelles; accession numbers KJ410682 and KJ410686; *N. oceanica* LAMB0001 draft organelles; accession numbers KJ410683 and KJ410687 ( http://www.ncbi.nlm.nih.gov/genbank). The transcript mapping data shown in Figure 6 is available in the NCBI Sequence Read Archive ( http://www.ncbi.nlm.nih.gov/bioproject/242770).

## Abbreviations

cpDNA: Chloroplast DNA; bp: base bairs; PDB: Protein data bank; ATP: Adenosine-5′-triphosphate; OSCP: Oligomycin-sensitivity conferring protein; ORF: Open reading frame; AAA: ATPases associated with various cellular activities; RMSD: Root mean square deviation.

## Competing interests

The authors declare that they have no competing interests.

## Authors’ contributions

SRS and RAC conceived the study. GR, SRS, RAC, and KK contributed to the manual curation of all annotations. SRS and KK completed the nucleotide and transcriptome analysis on the ATP synthase gene cluster. RJ generated and analyzed the secondary and tertiary structure predictions for AtpD, AtpG, and AtpA. RAC identified and analyzed the repeats. SRS completed the BLASTP analysis of each gene and completed the detailed analysis of RuBisCO and the Clp orthologs. KK, SRS, and GR constructed the pangenomes. ST completed the growth experiment and extracted RNA for transcriptomic analysis. GR completed the phylogenetic analysis of CbbX. OC completed the assembly of the *N. salina* chloroplast and mitochondrial genomes. GR, CM, MJ completed the sequencing and assembly of the *N. oculata* chloroplast and mitochondrial genomes. SRS, RAC, KK, GR, and RJ wrote the manuscript. All authors read and approved the final manuscript.

## Supplementary Material

Additional file 1: Figure S1 Local random docking of NS-AtpA-N terminus in the expected pocket of predicted NS-AtpD subunit. Plot of total energy (Rosetta Energy Units) vs RMS deviation of decoys for the selected model (Figure 7A) is indicated with an arrow (the minimum energy docked conformation corresponds with minimum RMS deviation from the selected model). Click here for file

Additional file 2: Figure S2 Alignment of the C terminus of RbcL. The residues highlighted in grey indicate the ‘tail’ region which interacts with the CbbX activase. Click here for file

Additional file 3: Figure S3 Protein alignment of ATP synthase subunit (AtpA). Click here for file

Additional file 4: Figure S4 Protein alignment of ATP synthse b’ subunit (AtpG). Click here for file

Additional file 5: Figure S5 Secondary structure prediction for the *N. salina* ATP synthase subunits using psipred. Secondary structure is denoted as H (helix), C (loops) and E (strands). The confidence of prediction ranges from 0 to 9. with 9 as high confidence and 0 as low confidence. Click here for file

Additional file 6: Figure S7 Template PDB structures used *for* modeling, (A) *E. coli* δ-subunit of F1FO ATP synthase (PDB code labv) used for NS-AtpD, (B) *S. cerevisiae* subunit G of V1VO ATPase (PDB code 2K88) used for NS-AtpG, (C) Uncharacterized protein BP1543 from *Bordetella pertusi tohama I* (PDB code 3KK4) used for NS-AtpA-N terminus. Click here for file

Additional file 7: Figure S6 Structural models of *N. salina* ATP synthase subunits. *Ab initio* structure prediction of (A) Ns-AtpD, (B) Ns-AtpA-Nterminus and (C) Ns-AtpG. Comparative Structural Models of (D) Ns-AtpD, (E) Ns-AtpA-N terminus, and (F) Ns-AtpG. Click here for file

Additional file 8 **Protein docking model of *N. salina *** **AtpD-A.** Click here for file

Additional file 9 **Protein model of the *N. salina *** **AtpG.** Click here for file

Additional file 10: Table S1 Inventory of *N. salina* Clp Homologs. Click here for file
