# Supplementary figures and images for: A pangenomic analysis of the Nannochloropsis organellar genomes reveals novel genetic variations in key metabolic genes
Source: BMC Genomics. 2014 Mar 19;15:212. doi: 10.1186/1471-2164-15-212 (PMC3999925; doi:10.1186/1471-2164-15-212)

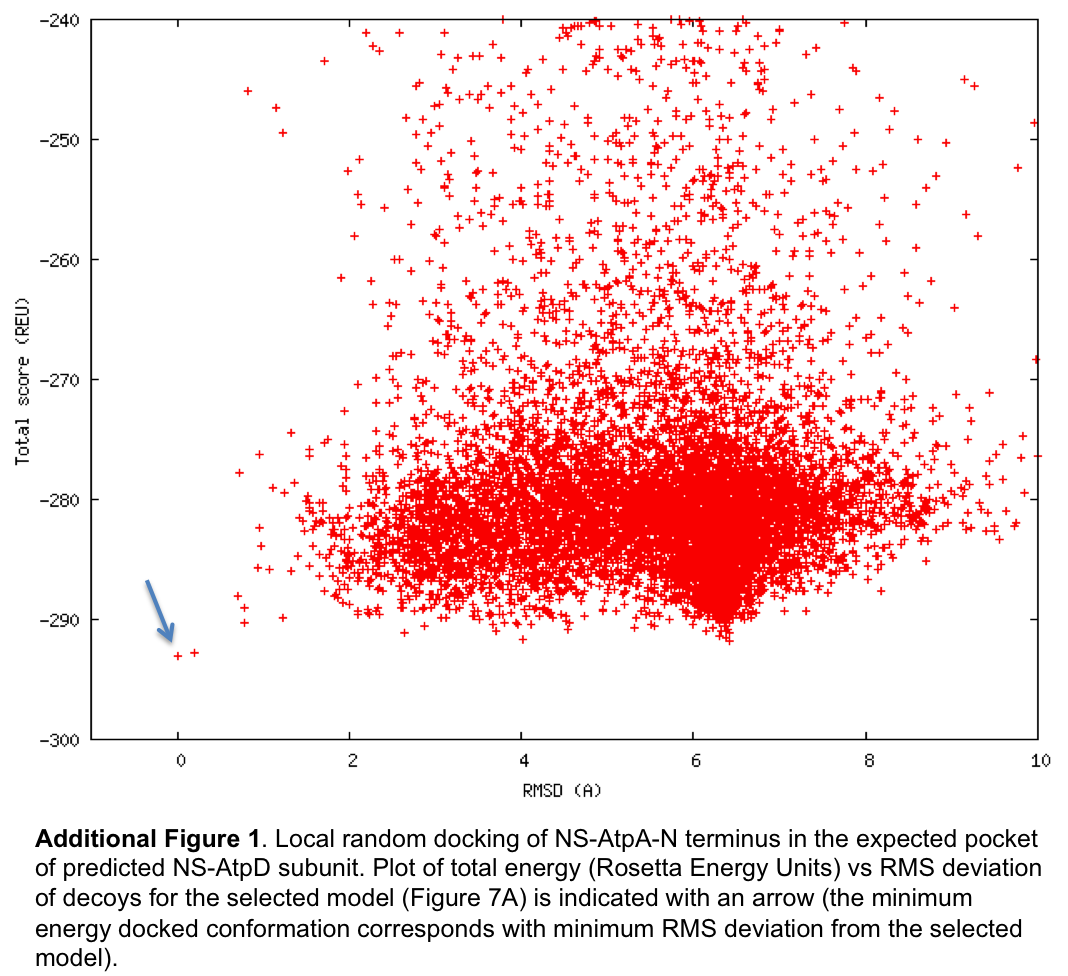

Supplement: Additional file 1: Figure S1 — Local random docking of NS-AtpA-N terminus in the expected pocket of predicted NS-AtpD subunit. Plot of total energy (Rosetta Energy Units) vs RMS deviation of decoys for the selected model (Figure 7A) is indicated with an arrow (the minimum energy docked conformation corresponds with minimum RMS deviation from the selected model). [file 1471-2164-15-212-S1.png]

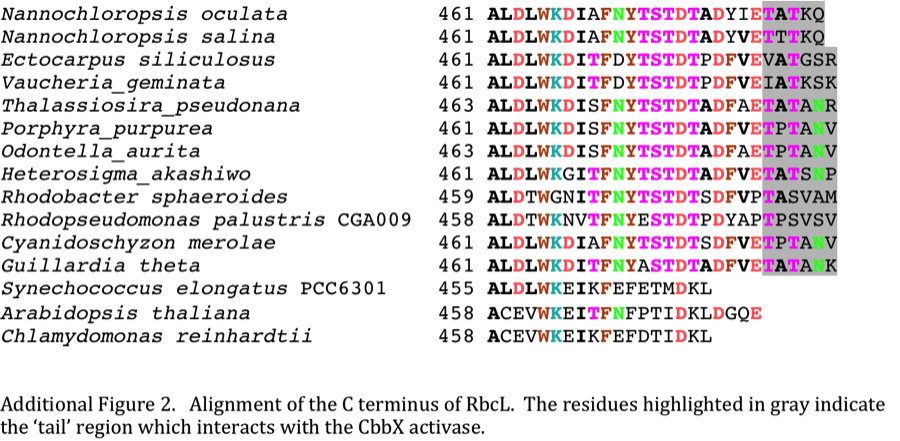

Supplement: Additional file 2: Figure S2 — Alignment of the C terminus of RbcL. The residues highlighted in grey indicate the ‘tail’ region which interacts with the CbbX activase. [file 1471-2164-15-212-S2.png]
