# Supplementary material for: A pangenomic analysis of the Nannochloropsis organellar genomes reveals novel genetic variations in key metabolic genes
Source: BMC Genomics. 2014 Mar 19;15:212. doi: 10.1186/1471-2164-15-212 (PMC3999925; doi:10.1186/1471-2164-15-212)
Supplement: Additional file 6: Figure S7 — Template PDB structures used for modeling, (A) E. coli δ-subunit of F1FO ATP synthase (PDB code labv) used for NS-AtpD, (B) S. cerevisiae subunit G of V1VO ATPase (PDB code 2K88) used for NS-AtpG, (C) Uncharacterized protein BP1543 from Bordetella pertusi tohama I (PDB code 3KK4) used for NS-AtpA-N terminus. [file 1471-2164-15-212-S6.pptx]

## Slide 1
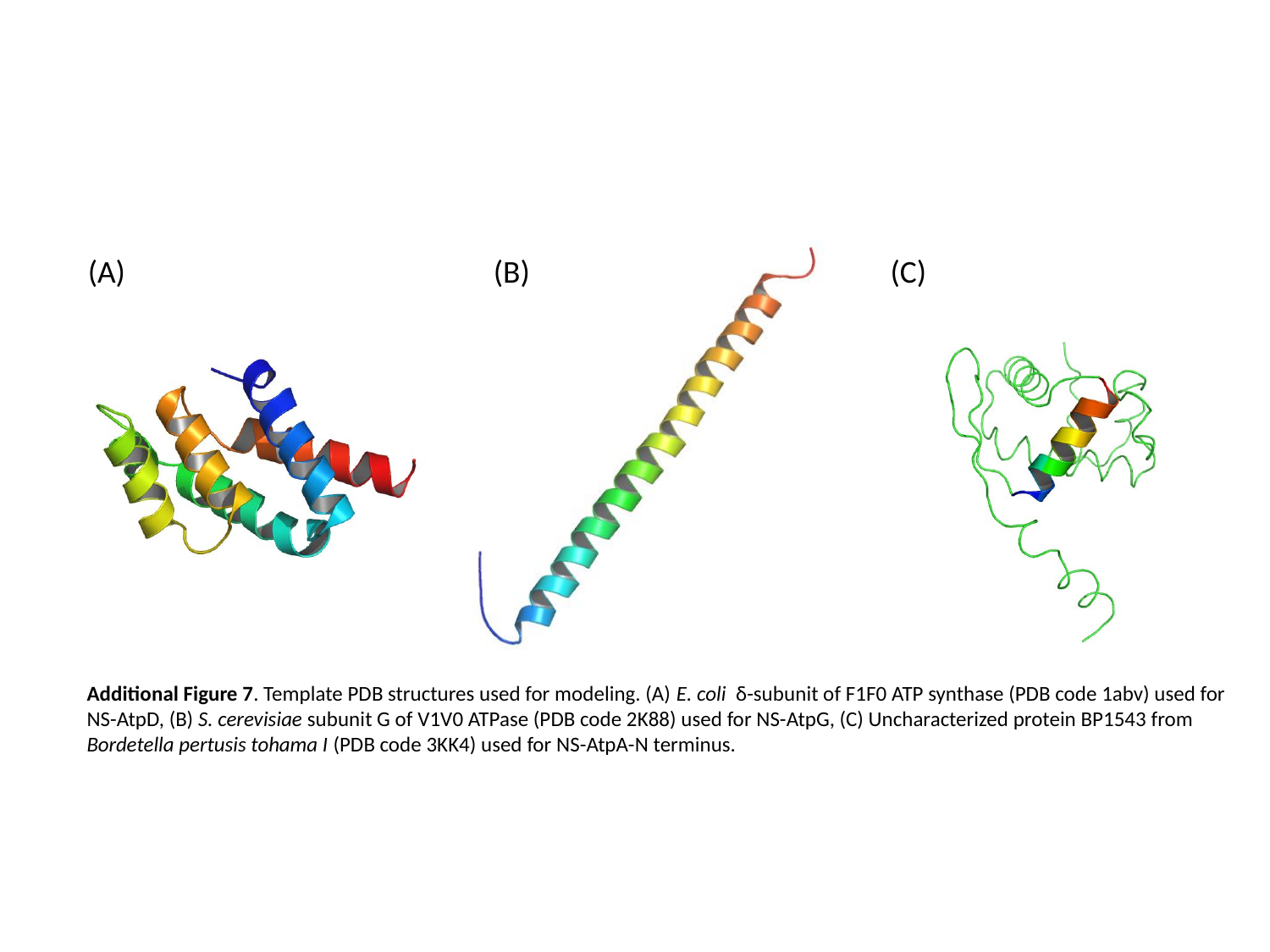

(A)
(B)
(C)
Additional Figure 7. Template PDB structures used for modeling. (A) E. coli δ-subunit of F1F0 ATP synthase (PDB code 1abv) used for NS-AtpD, (B) S. cerevisiae subunit G of V1V0 ATPase (PDB code 2K88) used for NS-AtpG, (C) Uncharacterized protein BP1543 from Bordetella pertusis tohama I (PDB code 3KK4) used for NS-AtpA-N terminus.
