# Supplementary material for: A pangenomic analysis of the Nannochloropsis organellar genomes reveals novel genetic variations in key metabolic genes
Source: BMC Genomics. 2014 Mar 19;15:212. doi: 10.1186/1471-2164-15-212 (PMC3999925; doi:10.1186/1471-2164-15-212)
Supplement: Additional file 10: Table S1 — Inventory of N. salina Clp Homologs. [file 1471-2164-15-212-S10.pdf]

Additional Table 1. Inventory of *N. salina* Clp Homologs.

Gene Name	Locus Tag	AA Length	Genome	Possible Function(s)	Closest Ortholog in Genbank –NR (Accession number; e-value)
clpN	Nsk_00142	149	Chloroplast	N-domain of Clp Chaperone	N-terminus of ClpA protease, [*Desulfobulbus propionicus* DSM 2032] (YP_004196194.1; 3.2)
clpC1	Nsk_00023	384	Chloroplast	D1-domain of Clp Complex; unfoldase	ATP-dependent Clp protease, [*Hydrogenivirga sp.* 128-5-R1-1] (ZP_02178006.1; 6e-106)
clpC2	Nsk_00076	449	Chloroplast	D2-domain of Clp Protease; protein stabilization	ATPase AAA-2 domain protein [*Arthrospira maxima* CS-328] (ZP_03275363.1; e-122)
clpP1	Nsk_5066	289	Nuclear	Protease	ATP-dependent Clp protease proteolytic subunit [*Ectocarpus siliculosus*] (CBJ27051.1; 2e-84)
clpP2	Nsk_2184	310	Nuclear	Protease	predicted protein [*Phaeodactylum tricornutum* CCAP 1055/1] (XP_002179995.1; 6e-73)
clpP3	Nsk_6949*	119	Nuclear	Protease	ATP-dependent Clp protease, proteolytic subunit ClpP [*Ectocarpus siliculosus*] (CBJ26066.1; 3e-62)
clpP4	Nsk_156	284	Nuclear	Protease	chloroplast clp protease P [*Ectocarpus siliculosus*] (CBN79074.1; 4e-131)
clpP5	Nsk_2067	228	Nuclear	Protease	catalytic subunit of clp protease [*Thalassiosira pseudonana* CCMP1335] (XP_002292583.1; 5e-110)
clpS1	Nsk_6320	194	Nuclear	Adaptor	predicted protein [*Thalassiosira pseudonana* CCMP1335] (XP_002287774; 8e-19)
clpS2	Nsk_5215	278	Nuclear	Adaptor	hypothetical protein THAOC_1439 [*Thalassiosira oceanica*] (EJK64828; 8e-12)

*partial gene
